# Quantitative sensory profiles of upper extremity chemotherapy induced peripheral neuropathy: Are there differences in sensory profiles for neuropathic versus nociceptive pain?

**DOI:** 10.1080/24740527.2019.1665992

**Published:** 2019-10-03

**Authors:** Elizabeth Andersen Hammond, Marshall Pitz, Pascal Lambert, Barbara Shay

**Affiliations:** a College of Rehabilitation Sciences, Rady Faculty of Health Sciences, University of Manitoba, Winnipeg, Manitoba, Canada; b Internal Medicine, Rady Faculty of Health Sciences, Medical Oncologist, University of Manitoba, Winnipeg, Manitoba, Canada; c Epidemiology and Cancer Registry, CancerCare Manitoba, Winnipeg, Manitoba, Canada

**Keywords:** neuropathic pain, chemotherapy-induced peripheral neuropathy (CIPN), quantitative sensory testing (QST), sensory neuropathy, S-LANSS

## Abstract

**Aims**: The aim of this study was to define the sensory phenotypes of taxane-induced peripheral neuropathy (TIPN) between neuropathic and nonneuropathic symptoms in a breast cancer population to identify future targets for mechanism-based pain management.

**Methods**: Participants (*n* = 48) with stage I–III breast cancer. Self-report questionnaires and quantitative sensory testing were used to assess sensory symptoms. The self-report version of the Leeds Assessment for Neuropathic Symptoms and Signs (S-LANSS) divided the groups into neuropathic and nonneuropathic sensory phenotypes. In total, five visits over approximately 8 months assessed each participant from pre-chemotherapy to 6 months post-chemotherapy.

**Results**: Out of 191 nerve assessments, 150 had an S-LANSS <12 defined as “nonneuropathic” and 41 scored >12, which was defined as “neuropathic.” Numeric Pain Rating Scale (NPRS) was analyzed based on percentages of those experiencing 1+ pain (graded 1/10 or higher) versus no pain. The neuropathic group had 82.9% of 1+ pain vs. 28.7% in the nonneuropathic group (odds ratio = 7.49; 95% confidence interval, 2.76–20.3; *P* = 0.001). The neuropathic group reported impaired function on the Disability of the Arm, Shoulder, and Hand (DASH) questionnaire (*P* = 0.002). Heat pain threshold resulted in statistical differences for the left hand but not the right hand in the neuropathic group (*P* = 0.05). No other quantitative data on warm/cool or cold or vibration demonstrated sensory differences between the groups.

**Conclusions**: Few differences in sensory profiles measured using quantitative sensory testing (QST) were found. Heat pain thresholds were normalized, possibly suggesting that the neuropathic group retained C-fiber and transient potential vanilloid 1 (TRPV1) function. Participants with neuropathic pain demonstrated significant differences with increased pain and decreased function.

## RÉSUMÉ

**Objectif**: Déterminer les phénotypes sensoriels associés à la neuropathie périphérique induite par le taxane dans une population de personnes atteintes de cancer du sein, selon qu’elles présentent des symptômes de neuropathie ou non, afin de déterminer les cibles futures pour la prise en charge de la douleur axée sur les mécanismes.

**Méthodes**: Participants (n = 48) atteints d’un cancer du sein de stade I-III. Des questionnaires d’auto-évaluation et des tests sensoriels quantitatifs ont été utilisés pour évaluer les symptômes sensoriels. À l’aide de la version d’auto-évaluation de l’outil d’évaluation des symptômes et des signes de la douleur neuropathique de Leeds (S-LANSS), les groupes ont été divisés en phénotypes sensoriels avec neuropathie et sans neuropathie. Au total, cinq visites échelonnées sur une période d’environ huit mois ont permis d’évaluer chaque participant avant le début de la chimiothérapie jusqu’à six mois après le début de celle-ci.

**Résultats**: 191 évaluations des nerfs, parmi lesquelles 150 participantes ont obtenu une note < 12 pour le S-LANSS, définis comme «sans neuropathie », tandis que 41 participantes ont obtenu une note > 12, définie comme « avec neuropathie ». L’échelle numérique d’évaluation de la douleur a été analysée sur la base du pourcentage de participantes éprouvant une douleur égale ou supérieure à 1 (note de 1/10 ou plus) comparativement à aucune douleur. 82,9 % des patientes du groupe avec neuropathie éprouvaient une douleur égale ou supérieure à 1 comparativement à 28,7% pour le groupe sans neuropathie (RR = 7,49, CI 95 % 2,76-20,3, *p* = 0,001). Le groupe avec neuropathie a fait état d’une altération du fonctionnement selon le questionnaire DASH (*p* = 0,002). Des différences significatives ont été observées en ce qui concerne le seuil de la douleur thermique pour la main gauche, mais pas pour la main droite dans le groupe avec neuropathie (p = 0,05). Aucune autre donnée quantitative portant sur la sensibilité aux températures chaudes, tièdes ou froides, ou encore à la vibration, n’a révélé de différences sensorielles entre les groupes.

**Conclusions**: On a constaté peu de différences entre les profils sensoriels mesurés par les tests sensoriels qualitatifs. Les seuils de douleur thermique ont été normalisés, ce qui indique probablement le maintien du fonctionnement des fibres de C et du TRPV1 chez le groupe avec neuropathie. Des différences significatives ont été observées chez les personnes souffrant de douleur neuropathique, dont une augmentation de la douleur et une diminution du fonctionnement.

## Introduction

Taxane chemotherapy is used by oncologists for many solid tumors, including in patients with stage I–III breast cancer treated with curative intent. Eighty to 97% of patients exposed to taxane chemotherapy will experience a sensory neuropathy in the hands and/or feet caused by the neurotoxic medication.^1,2^ Persistence of symptoms for many exposed to taxanes can be for months or years posttreatment.^2–4^ Neuropathic pain is difficult to treat, and presently there are few effective treatment options specific to chemotherapy-induced peripheral neuropathy (CIPN).^4,5^ Duloxetine is the only first-line agent with moderate success for CIPN.^5,6^ Unfortunately, side effects including sedation, nausea, constipation, and ataxia limit the usefulness of this drug.^7^ Though some patients experience primarily sensory loss (hypoesthesia), others have burning neuropathic pain (hyperalgesia and allodynia) or various combinations of positive and negative sensory profiles, including hypoesthesia, dysesthesia, hyperalgesia, and allodynia. Reviews on CIPN frequently describe all symptoms of the neuropathy (both positive and negative) together.^2,8–10^ There are substantial differences in quality of life for breast cancer survivors between experiencing “a little numbness” and “burning pain,” and it is important to separate and define these two phenotypes. Mechanism-based pain management theory suggests that treatment of neuropathic pain should be specific to the sensory phenotype rather than the diagnosis. It is theorized that specific pharmacological treatments may respond more appropriately to specific phenotypes (i.e., heat hyperalgesia, cold allodynia, or lower pain pressure thresholds). For example, patients with postherpetic neuralgia with higher heat pain thresholds benefited more from opioids than those with lower heat thresholds.^11,12^ A mechanism-based pain management approach recognizes that, at present, it remains unknown which medications work on specific sensory signs. Large multicenter trials using quantitative sensory testing (QST) have been recommended to identify similar sensory characteristics underlying different causes of neuropathic pain.^13^

This descriptive study aimed to gain a better understanding of the different sensory phenotypes experienced in taxane-induced peripheral neuropathy (TIPN). The goal was to determine whether different sensory characteristics were evident using QST in participants with and without neuropathic pain.

### Aims

The aim of this study was to define the sensory phenotypes of TIPN in a breast cancer population. The self-report version of the Leeds Assessment of Neuropathic Symptoms and Signs (S-LANSS) was used to define symptoms that were “neuropathic” versus “nonneuropathic” during and after chemotherapy. Using QST, we sought to improve understanding of neuropathic and nonneuropathic pain profiles in order to identify future targets for mechanism-based pain management in patients with breast cancer.

### Hypothesis

It was hypothesized that the S-LANSS questionnaire would identify between-group differences in QST measures for neuropathic and nonneuropathic symptom profiles. Specifically, the neuropathic pain group would have lower pain pressure thresholds (central sensitization), lower thermal detection thresholds (improved perception), and lower cold pain thresholds demonstrating hyperalgesia. It was also hypothesized that participants in the neuropathic group would report higher pain scores and decreased upper extremity function.

## Methods

### Data collection

Participants’ data were collected as part of a larger physical therapy and nerve health trial evaluating the effects of a home exercise program on upper extremity pain and nerve function. Informed written consent was obtained from each participant prior to participation. Forty-eight participants completed self-report questionnaires and quantitative sensory testing as part of the trial and were seen for reassessment of nerve function during and after chemotherapy on four occasions over a 7- to 8-month period. Nerve reassessments were completed at the Pain Research Laboratory, College of Rehabilitation Sciences, University of Manitoba. Reassessments occurred (1) midway through chemotherapy, (2) at the end of chemotherapy, (3) 3 months post-chemotherapy, and (4) 6 months post-chemotherapy. Ethics approval was granted by both the Health Research Ethics Board (H:2014:281) at the University of Manitoba and the Research Resource Impact Committee (RRIC 2014–031) at CancerCare Manitoba. Clinical trials registration number NCT02239601.

The S-LANSS defined neuropathic pain.^14^ The scale ranges from 0 to 19, with a score above 12 indicative of neuropathic pain/symptoms. Studies using the S-LANSS have demonstrated good accuracy and specificity in a cancer population.^15,16^ Scores >12 defined the neuropathic group and scores <12 defined the nonneuropathic group. Participants with stage I–III breast cancer being treated with taxane chemotherapy, either TC (docetaxel 75 mg/m^2^ and cyclophosphamide ×4) or FECD (5-fluorouracil, epirubicin, cyclophosphamide ×3, followed by docetaxel 100 mg/m^2^ ×3), were included. All quantitative outcome measures, functional measures, and pain questions were specific to assessment of the hands.

### Outcome measures

Thermal detection thresholds (warm and cool) and thermal pain thresholds (hot and cold) measure Aδ and C-fiber function and were used to quantify the neuropathy on each assessment. The Neurosensory Analyzer (TSA II, Medoc, Israel) 30-mm thermode was attached to the palmar surface of the distal phalanx of the index and middle fingers. Temperature was increased or decreased by 0.1°C increments until the participant pressed a button indicating temperature detection thresholds or thermal pain. The temperature immediately returned to baseline (32°C) once the button was pressed. The participant was always in control and was never at risk for tissue damage (temperature limits are set to vary from 0°C to 50°C).

The TSAII Vibration Sensory Analyzer module for the Medoc was used to test vibration perception involving Aβ nerve fibers. The palmar distal phalanx of the index finger lightly touched the sensor. Different, random, and varying vibration amplitudes (0–130µm at 0.1–4.0 microns/second) using the limits testing method on the VSA were delivered with the participant responding “yes/no” to sensing the vibration. Vibration perception was selected because it has been suggested to be the first clinical sign of CIPN symptoms.^17^

Pressure algometry measured pressure/pain thresholds (Somedic AB, Sweden). The left quadriceps muscle (distant site from the source of CIPN pain) was tested as a measure of relative hyperalgesia. Increasing pressure was applied to the left quadriceps until sensation changed from a feeling of pressure to a feeling of pain. The participant pressed a button and the test stopped when pain was perceived. Force in kilopascals was recorded.

The Numeric Pain Rating Scale (NPRS) is an 11-point scale rating hand CIPN pain (0–10) on each assessment visit. The scale categories range from *no pain at all* (0) to *the worst pain imaginable* (10).

Disability of the Arm, Shoulder, and Hand (DASH) was used to gauge upper/lower limb function. The DASH, a self-report questionnaire, was chosen due to its high test–retest reliability and the responsiveness and construct validity in breast cancer patients compared to other quality of life measures.^18,19^ The minimal clinically important difference is a change score of 15.

### Data analysis

Outcomes were compared over time between participants with either an S-LANSS score ≥12 or <12 (i.e., S-LANSS was included as a time-varying predictor). The thermal and vibration QST data assessed possible sensory differences between the surgical and nonsurgical sides. Mixed models were used to compare outcomes between groups, to account for repeated measurements and unequal time intervals. Linear mixed models were used to predict continuous outcomes when the assumption of normality was met. Quantile mixed models were used to predict ordinal outcomes or continuous outcomes where the assumption of normality was not met. Logistic mixed models were used for predicting binary outcomes, and the results were marginalized using the approach by Hedeker et al.,^20^ which converts subject-specific estimates to population-averaged estimates. Residual plots were used to evaluate the assumption of normality and to detect outliers. The assumption of linearity for continuous predictors was evaluated using restricted cubic splines. Analyzes were run using R version 3.4.1 and SAS version 9.4.^21^

## Results

A total of 191 participant visits were included in this analysis. Table 1 shows the participant demographics including age, cancer stage, cancer side, type of surgery, adjuvant treatment, history of nerve entrapment or damage, and loss of shoulder range of motion (ROM) at baseline. Participants had a mean age of 61.5 (range 34–78). Seventeen (35.4%) participants were classified as stage I, 22 (45.8%) as stage II, and 9 (18.8%) as stage III. Nineteen (39.6) participants had right-sided breast cancer, 28 (58.3%) had left-sided breast cancer, and 1 (2.1%) had bilateral cancer. Ten (20.8%) participants received reconstructive surgery and 37 (77.1%) received radiation post-chemotherapy. Thirty (62.5%) participants received FECD and 18 (37.5%) received TC.

**Table 1.** Demographics.

Participant demographics	
Age, mean (SD)	61.5 (23.33)
Stage, *n* (%)	
I	17 (35.40)
II	22 (45.80)
III	9 (18.80)
Cancer side, *n* (%)	
Right	19 (39.60)
Left	28 (58.30)
Bilateral	1 (2.10)
Surgery, *n* (%)	
Lumpectomy	27 (56.20)
Single mastectomy	15 (31.30)
Bilateral mastectomy	6 (12.50)
Reconstruction	10 (20.80)
Radiation, *n* (%)	37 (77.10)
Docetaxel, *n* (%)	
FECD (six rounds, three include taxane)	30 (62.50)
TC (four rounds, all include taxane)	18 (37.50)
Loss of range of motion–shoulder, *n* (%)	13 (27.10)
History of upper extremity nerve problems, *n* (%)	13 (27.10)

FECD = 5-fluorouracil, epirubicin, cyclophosphamide ×3, followed by docetaxel 100 mg/m^2^ ×3; TC = docetaxel 75 mg/m^2^ and cyclophosphamide ×4.

From the regression model, the DASH score showed statistically significant differences between the groups in that the neuropathic group demonstrated impaired upper limb function (*P* = 0.002). More important, the DASH score mean of 35 (31–46) in the nonneuropathic group versus 51 (41–67) in the neuropathic group met the minimum clinical important difference in change scores, indicating that these groups are not only statistically but also clinically different.^18^

Due to the heavily skewed distribution of pain scores, the 11-point NPRS was analyzed based on percentages of those experiencing pain (NPRS 1 or higher) versus no pain. Using logistic mixed models predicting binary outcomes, the proportion of participants rating pain was 82.9% in the neuropathic group versus 28.7% in the nonneuropathic group. This was statistically significant and reflects the distinct differences in pain symptoms between the groups (odds ratio= 7.49; 95% confidence interval, 2.76–20.3; *P* = 0.001) for the hands.

Comparing neuropathic to nonneuropathic profiles, heat pain threshold resulted in statistical differences between the groups for the left hand (*P* = 0.05). Values for the neuropathic group (left 42.3°C, right 42.5°C) were closer to age-matched normative values (41.8°C–42.1°C) than the nonneuropathic group values (left 43.7°C, right 44.1°C).^22^ All other variables including warm and cool detection, cold pain, vibration sensation, and pain pressure algometry were not significantly different between the groups. No sensory differences were found comparing thermal detection, thermal pain thresholds, or vibration perception between the surgical and nonsurgical sides.

**Figure 1.**
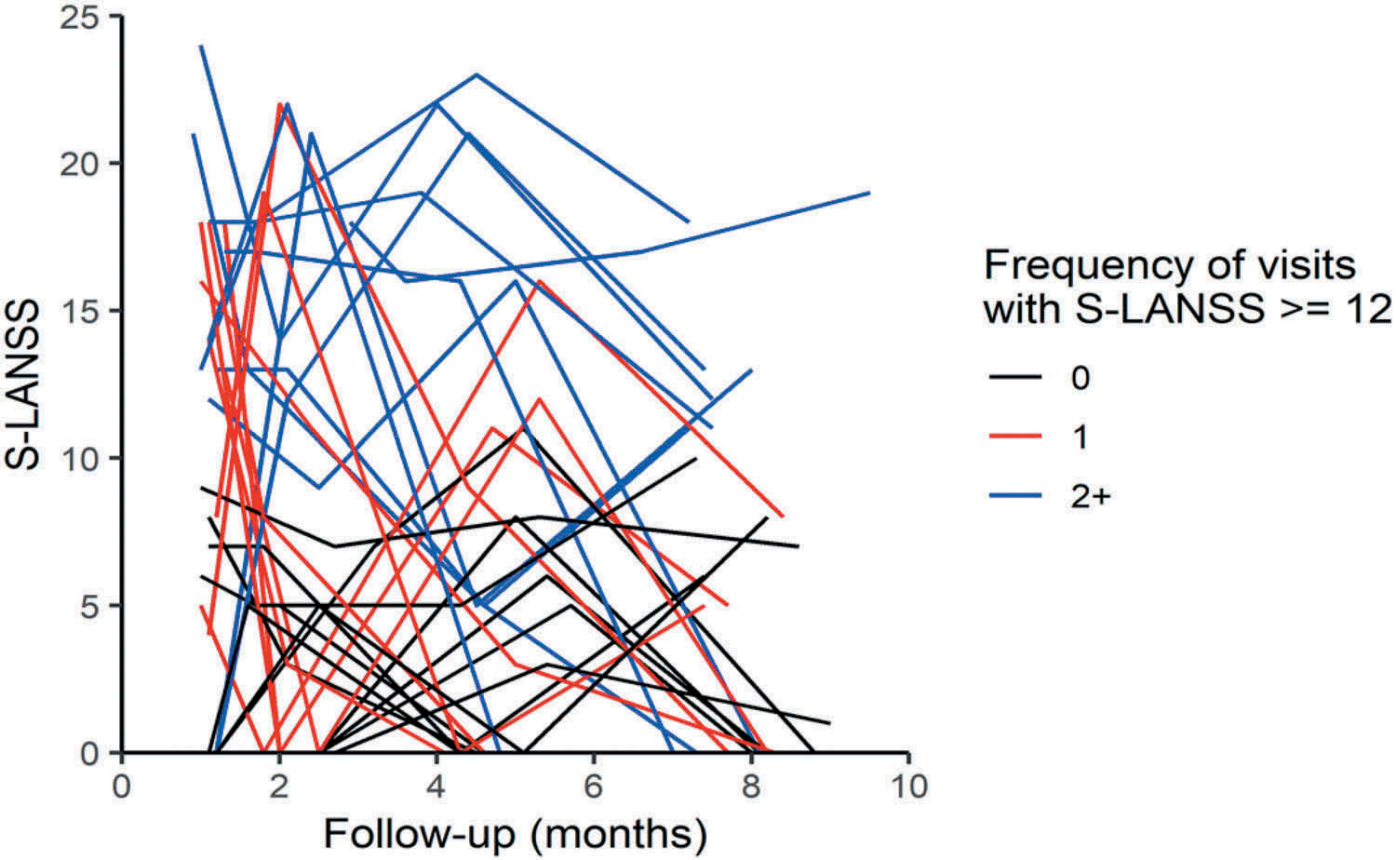
Distribution of S-LANSS scores over time. This graph represents each participant’s S-LANSS score over time. Participants with no neuropathic pain are shown in black. Participants indicating neuropathic pain on one visit only are in red. When neuropathic symptoms are transient, symptoms are more likely to be present during chemotherapy and decrease over time. Participants who reported neuropathic pain on multiple visits are shown in blue. Participants reporting neuropathic pain symptoms on multiple visits were likely to have persistent symptoms at the end of the trial.

Out of 191 nerve assessments, 150 had an S-LANSS <12, defined as nonneuropathic, and 41 scored >12, which was defined as neuropathic. Therefore, 21% of participant nerve assessment visits demonstrated neuropathic pain. The remaining 79% either had no complaints of CIPN sensory symptoms or no pain defined as neuropathic associated with CIPN dysesthesias. Participants with transient neuropathic pain (reported on one visit only) were more likely to experience symptoms during chemotherapy, and participants who rated neuropathic pain on more than one visit were also likely to experience persistent neuropathic symptoms at the end of the trial. Figure 1 plots all participants’ S-LANSS scores over time. Though 21% of nerve assessments indicated neuropathic pain, only 10.4% of participants (*n* = 5) continued to suffer with persistent neuropathic pain at the end of the trial (6 months post-chemotherapy). Table 2 shows the mean (SD) and median (Q1–Q3) outcome measures with *P* values for the participants divided into neuropathic and nonneuropathic conditions.

**Table 2.** Neuropathic and nonneuropathic symptom profiles.

	Neuropathic	Nonneuropathic	*P* value
Warm detection mean (SD)			
Left	35.7 (2.38)	35.3 (2.22)	0.27
Right	36.3 (1.60)	35.9 (1.90)	0.65
Cool detection mean (SD)			
Left	29.8 (1.69)	30.0 (1.67)	0.35
Right	29.3 (1.94)	29.3 (2.86)	0.35
Hot pain thresholds mean (SD)			
Left	42.3 (3.33)	43.7 (3.51)	0.17
Right	42.5 (2.93)	44.1 (3.11)	0.05*
Cold pain thresholds mean (SD)			
Left	16.8 (9.19)	14.2 (8.97)	0.41
Right	16.5 (8.79)	13.9 (9.00)	0.46
Vibration median (Q1–Q3)			
Left	0.14 (0.04–0.24)	0.14 (0.04–0.34)	0.38
Right	0.23 (0.13–0.49)	0.23 (0.13–0.41)	0.76
Pressure mean (SD)	752 (315)	843 (348)	0.52
NPRS, *n* (%)			
No pain	7 (17.1)	107 (71.3)	
Pain 1+	34 (82.9)	43 (28.7)	0.001*
DASH, median (Q1–Q3)	51 (41–67)	35 (31–46)	0.002*
Number of visits with neuropathic pain, median (interquartile rage)			
2+	13.5 (10.5–18.0)		0.001*
1	3.5 (0–11.3)		0.001*
0	0 (0–0)		

Q1–Q3 = interquartile range representing the 25-75 percentile; NPRS = Numeric Pain Rating Scale; DASH = Disability of the Arm, Shoulder, and Hand.

* statistically significant.

## Discussion

The S-LANSS is an easy-to-administer, quick, and useful tool to evaluate neuropathic symptoms in a breast cancer population. The S-LANSS clearly delineated neuropathic pain, indicating significantly higher pain and impaired upper extremity function between the neuropathic and nonneuropathic CIPN groups. In addition to increased pain and loss of function, severity of CIPN symptoms has been associated with depression, anxiety, and poor sleep quality.^23^ Costs to patients and the health care system from CIPN have been quantified at over $17 000 more per year compared to patients without CIPN.^24^

Results of the QST data revealed significant differences in left hand heat pain thresholds, with the neuropathic pain group demonstrating lower heat pain thresholds when compared to the nonneuropathic group. A potential explanation for these findings is preservation of C-fiber and Aδ fiber function. The lower heat pain thresholds in the neuropathic group (left 42.3°C, right 42.5°C) are closer to those of normative age-matched values (41.8°C–42.1°C) than the nonneuropathic group values (left 43.7°C, right 44.1°C). The nonneuropathic group demonstrated slightly higher thresholds than age-matched normative data, possibly suggesting heat hypoesthesia.^22^ Reviews have identified heat hypoalgesia with cold allodynia as a characteristic of painful CIPN.^10,25^ Suggested mechanisms for heat hypoesthesia are the loss of Aδ and C-fibers and transient potential vanilloid 1 (TRPV1) receptors.^25^ Heat pain thresholds similar to normative age-matched values for the neuropathic group may thus suggest preservation of these receptors and pathways. Transient receptor potential channels (TRPs), specifically TRPV1, are important in pain transmission directly through Ca^2^+ signaling and activation of second messengers.^26^ Given the importance of sensitization and TRPs in pain signaling, a lack of correlation between heat hypoesthesia and the neuropathic pain group may be expected. An alternate explanation is that a significant finding only for the hand may be due to a random effect of assessing multiple variables rather than preservation of sensory fibers.

Cold allodynia (commonly associated with neuropathic pain) and reduced pain pressure thresholds (suggested to represent relative hyperalgesia) demonstrated no significant between-group differences. This was surprising and unexpected; however, it is possible that the mechanisms causing CIPN (microtubule stability at the distal axon) do not result in different sensory profiles sensitive to QST measurement. Neuropathic pain is thought to be initiated and maintained, at least partly, by the immune system. Microglia and mast cell activation along with the recruitment of astrocytes incite and maintain neuroinflammation.^27–30^ Neuroimmune changes may not primarily affect the cutaneous receptors that are involved in QST measurement. Neuropathic pain may primarily impact higher order processing and interpretation of pain in the central nervous system.

As expected, the neuropathic group had statistically significant higher reports of pain (NPRS) and decreased function as reported by the DASH. This presentation is often witnessed clinically. Significant pain often extending beyond the site of injury, poor function, reduced work and activity levels, and disrupted sleep are common features of neuropathic pain. Neuropathic pain symptoms correlate with higher medical comorbidities, higher reported pain intensity, and greater disability burden than other causes of pain.^31–33^ This often results in poorer prognosis, and studies have identified higher levels of depression and disability.^34,35^ Pain catastrophizing and pain acceptance are important in treatment outcomes, and a multidisciplinary focus has been suggested for this population.^36^

Large individual variability exists in the location and intensity of neuropathic symptoms. Cumulative dose, schedule, combination therapy, preexisting risk factors (including diabetes, advanced age, smoking, increased alcohol consumption), increased body mass index, and genetic predisposition have been used to explain this variability.^37–40^ Our study controlled for some variability by using a single cancer type, single drug, similar dose and schedule, as well as a consistent time frame for repeat nerve assessments. Other factors such as smoking, body mass index, and alcohol consumption were not included in the current study.

Our data revealed that 21% of nerve assessments indicated pain neuropathic in origin. As Figure 1 shows, most of the reports of neuropathic pain were transient during chemotherapy (*n* = 16). Ten participants reported Neuropathic pain on multiple visits. Half of these participants (*n* = 5) or 10.4% continued to suffer with persistent neuropathic pain at the end of the trial (6 months post-chemotherapy). As clinicians, it is important to recognize that neuropathic pain during chemotherapy is expected to be transient (red lines in Figure 1). Symptoms that persist past the end of chemotherapy should be addressed as soon as possible because these are likely to remain past 6 months post-chemotherapy (blue lines in Figure 1). Our final results at 6 months post-chemotherapy are consistent with epidemiological data that identify 10% of the general population as experiencing pain that is neuropathic in nature.^41^ Systematic reviews identifying the prevalence of neuropathic pain specific to cancer patients report estimates between 20% and 40%.^42^ These numbers are consistent with our findings that 21% of assessments were defined as neuropathic pain throughout the trial. Systematic reviews on neuropathic pain estimates specific to breast cancer identify pooled estimates ranging from 14.2% to 57.1%.^43^ It is possible that the variability in prevalence reflects data collection at different time points (i.e., during chemotherapy or after treatment).

Preexisting neuropathy or prior history of nerve injury (including entrapment such as carpal tunnel) has been identified as a potential risk factor for CIPN.^8,10,44^ Out of the five participants who had persistent neuropathic pain at the end of the trial, three began chemotherapy with restricted ROM and two had a history of upper extremity nerve injury. This may be important, contributing to a dual nerve disorder. The double crush syndrome or dual nerve disorder, as it is now called, was first hypothesized by Upton and McComas in 1973.^44^ It states that “axons that have been compressed at one site become especially susceptible to damage at another site” (361).^45^ It is thought that a minimal amount of nerve compression affects axoplasmic flow but is below the threshold for clinical symptoms. A secondary insult further reduces axoplasmic flow, resulting in clinical symptoms and denervation. Expert views on the dual nerve disorder theory^46^ agreed that neurotoxic medication when combined with other nerve disorders might make the nervous system more susceptible to damage. This may be similar to the dual nerve disorders frequently seen in patients with diabetes where the metabolic damage to the peripheral nervous system compounds the effects of entrapment neuropathies.^47^

Unfortunately, this current descriptive study did not find definitive associations in QST to support the mechanism-based approach. The mechanism-based approach focuses primarily on a biomedical explanation. Pain, especially chronic and neuropathic pain, is known to have a substantial psychosocial component that needs to be integrated with the biological mechanisms. The current study did not measure psychosocial components of CIPN pain (for example; measuring pain catastrophizing, anxiety, or fear). Despite this, it is important to understand that different sensory complaints in CIPN do not seem to have distinct sensory profiles as measured by QST with the possible exception of heat pain thresholds.

The S-LANSS can quickly evaluate sensory phenotypes of neuropathic pain impacting quality of life and is useful in a clinic setting. An NRPS alone cannot distinguish between neuropathic and other nociceptive pain. The S-LANSS is easy and quick to administer, has good accuracy and specificity in a cancer population,^15,16^ and is a good screening tool to identify potential neuropathic symptoms from CIPN.

### Limitations

This data came from a larger physical therapy study evaluating a home program aiming to improve CIPN symptoms with ROM and nerve mobility exercises that would also help restore function postoperatively, minimizing a dual nerve disorder in the upper limb. Though it is recognized that CIPN is a symmetric, bilateral sensory neuropathy affecting both hands and feet, our study only included the hands, for a few reasons. Doubling the time at reassessment to assess both hands and feet was not feasible (midway though and at the end of chemotherapy). We were also concerned with the risk of attrition if the home physical therapy program took too much time to complete. The upper limb was chosen because of the known upper extremity morbidity in this population.^44,48,49^ Nevertheless, it is recognized that some symptoms were overlooked by excluding the lower limb. There is also the possibility that the heat pain threshold data were a random association from analysis including multiple comparisons, especially considering that statistical significance was not bilateral. Future studies should use the visual analog scale as a continuous measure in place of the NPRS for improved sensitivity. Though the purpose was to evaluate specific sensory phenotypes for mechanism-based pain management, the psychosocial aspects of pain were not assessed. Finally, QST presents a quantifiable stimulus but with a subjective response. Attention, focus, and standardized assessment all impact the validity and reliability of QST. For the purposes of the present study, QST provided the most appropriate and descriptive data for quantifying small fiber sensory neuropathy.

## Conclusion

Currently, the causes of both CIPN and neuropathic pain are not established. Both CIPN and neuropathic pain conditions are difficult to treat with few effective options. Why some patients with breast cancer treated with chemotherapy report severe painful sensory disturbances whereas others report a slight amount of numbness remains unknown. It is also unclear whether normal heat thresholds may be part of the neuropathic pain symptom profile (suggesting intact C-fibers and TRPV1 receptors that transmit pain). The neuropathic pain group may be predisposed to more severe symptoms due to decreased upper extremity ROM and prior nerve damage (the dual nerve disorder hypothesis). Further research is required to determine whether peripheral neuro-inflammation, processing at the spinal cord or higher order centers, or other possible mechanisms need to be targeted for effective therapy.
